# Left ventricular mechanics analysis after endocardial radiofrequency ablation for hypertrophic obstructive cardiomyopathy

**DOI:** 10.1016/j.hrcr.2024.11.009

**Published:** 2024-11-17

**Authors:** Julieta Morales-Portano, Martin Ortiz-Avalos, Gerardo Rodríguez-Diez, Elias Noel Andrade-Cuellar, Jorge Lara-Vargas, Mani Vannan

**Affiliations:** 1 Departments of Electrophysiology and Cardiology, Centro Médico Nacional “20 de Noviembre”, ISSSTE, Mexico City, Mexico; 2 Cardiovascular Imaging, Piedmont Heart Institute, Piedmont Atlanta Hospital, Atlanta, GA

**Keywords:** Radiofrequency ablation, Hypertrophic obstructive cardiomyopathy, LVOT gradient, Septal ablation, Electroanatomical mapping, Myocardial strain

**Key Teaching Points**

• ERASH exerts a therapeutic effect on local transmural myocardial contraction, specifically in the basal septal region. Basal septal regional strain may serve as a predictor of short-term left ventricular remodeling, which is associated with a reduction in left ventricular outflow tract (LVOT) obstruction.
• ERASH induces a shift in myocardial contraction mechanics, characterized by a decrease in longitudinal strain and a compensatory increase in circumferential strain.
• Long-term outcomes of ERASH are less predictable; close monitoring of myocardial strain and LVOT gradients is essential for the early detection of recurrences, which may necessitate further interventions or adjustments in treatment.

## Introduction

Hypertrophic obstructive cardiomyopathy (HOCM) is a significant cause of sudden cardiac death and heart failure.^1^^,^^2^ Approximately 8% of patients are not suitable candidates for septal alcohol ablation (SAA).^3^ When myectomy is not feasible, endocardial radiofrequency ablation of septal hypertrophy (ERASH) has proved effective in reducing left ventricular outflow tract (LVOT) gradients by inducing local contraction impairment without significantly reducing septal mass. The long-term impact of ERASH on LV mechanics, however, is still not well established.^4^ In this case series, we report 3 cases in which ERASH was guided by 3D electroanatomic mapping and intracardiac echocardiography (ICE).

## Case reports

### Case 1

**Figure 1:**
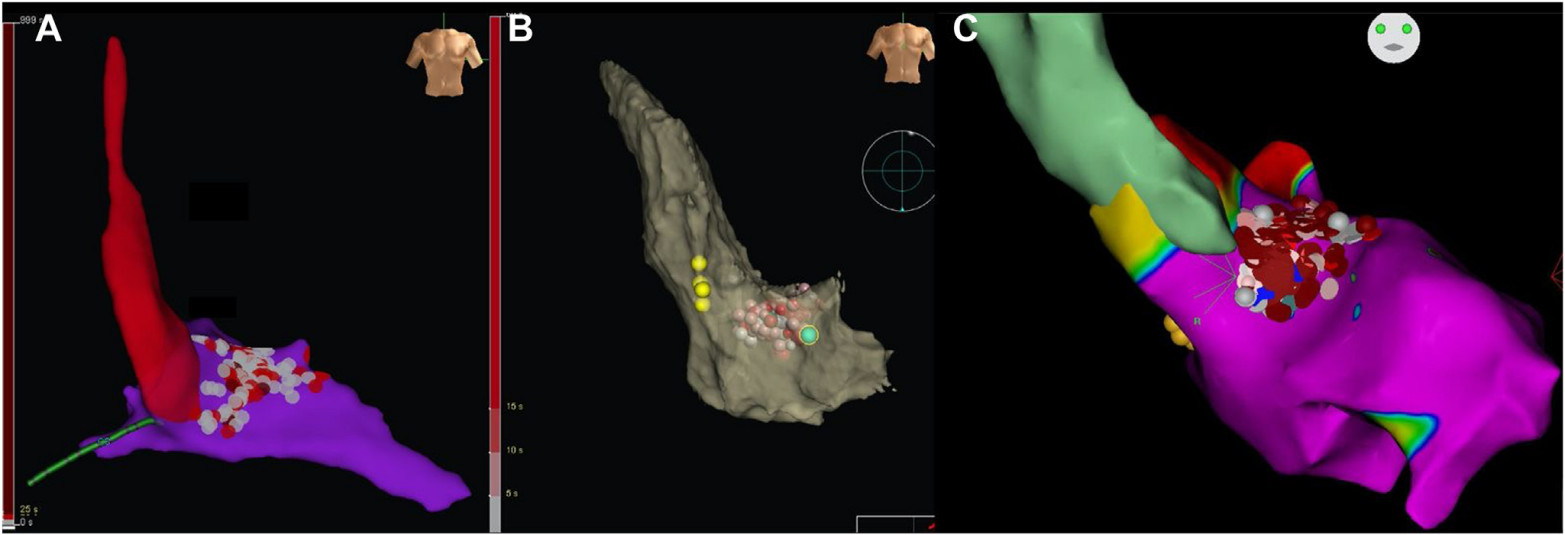
3D-guided anatomic map of LV and radiofrequency ablation lesions (*gray and red dots*) in an anteroposterior view. **A** and **C:** Cases 1 and 3: Anteroposterior view, EnSite Precision™ cardiac mapping system. **B:** Case 2: 3-D CARTO anteroposterior view with their corresponding pressure tracings. The Radiofrequency lesions (*red and gray dots*) are delivered in the area of the gradient, away from the conduction system (*yellow dots*).

**Figure 2:**
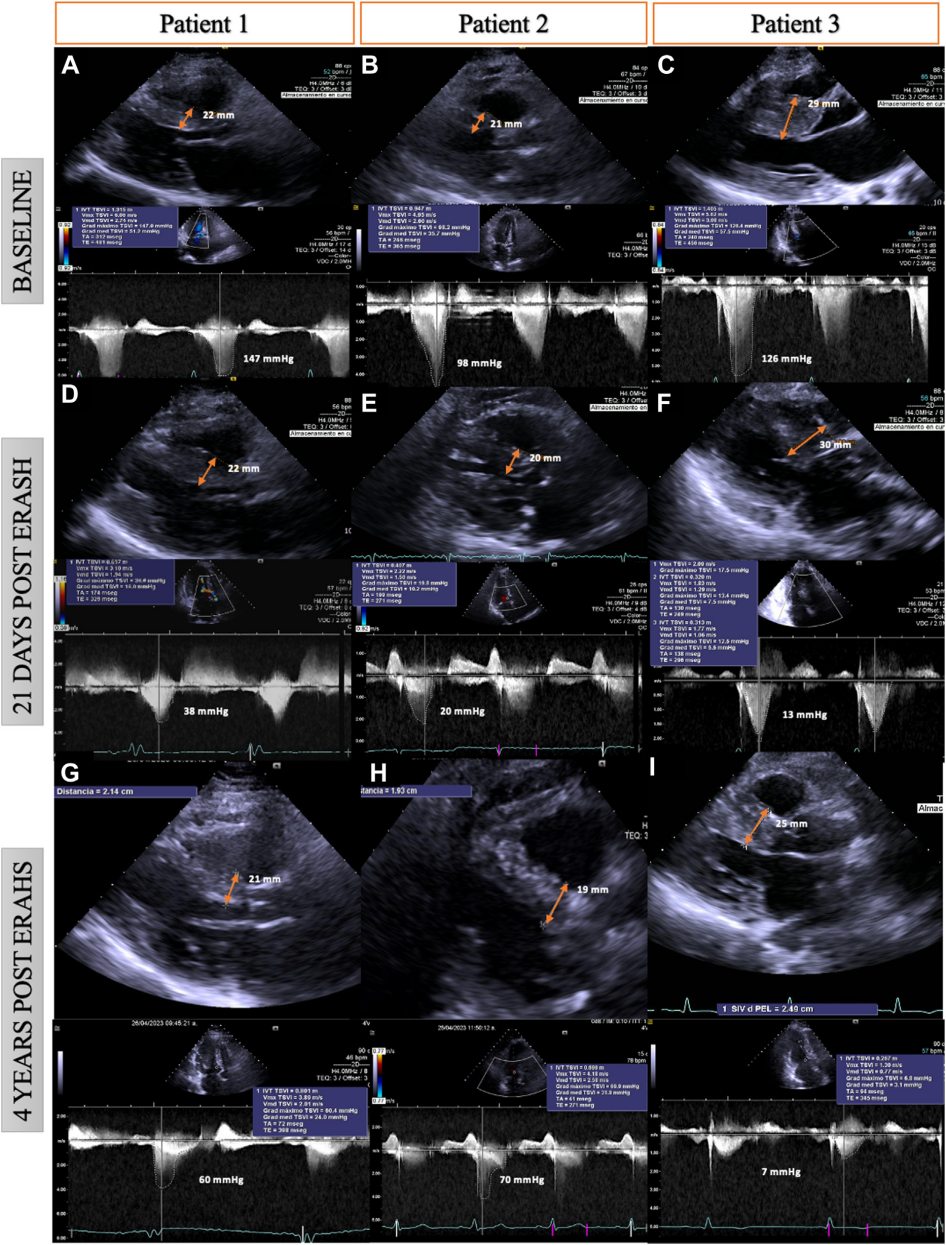
Echocardiographic measurements of septal thickness and LVOT gradients performed under resting conditions before ERASH (**A:** Case 1; **B:** Case 2; **C:** Case 3), at 21 days’ follow-up (**D:** Case 1; **E:** Case 2; **F:** Case 3) and 4 years post-ERASH (**G:** Case 1; **H:** Case 2; **I:** Case 3). ERASH = endocardial radiofrequency ablation of septal hypertrophy; LVOT = left ventricular outflow tract.

A 39-year-old woman had New York Heart Association (NYHA) III dyspnea, family history of HOCM in her grandfather and 2 uncles, and a previous episode of aborted sudden cardiac death. She was treated with beta-blockers and disopyramide, but a prior SAA had failed. An implantable cardioverter-defibrillator was placed for secondary prevention. The electrocardiogram (ECG) showed left ventricular hypertrophy with a Sokolow score of 36 and a Cornell score of 28. Transthoracic echocardiography (TTE) showed normal left ventricular ejection fraction (LVEF), maximal interventricular septal wall thickness (IVS WT) of 22 mm, and an LVOT gradient of 147 mm Hg at rest. ERASH was performed using the EnSite Precision™ 3D mapping system (Abbott, Abbott Park, IL), delivering 50 W for 30 seconds per lesion in the basal and mid-interventricular septal wall (Figure 1A). Noncapture of pacing was confirmed during the procedure to ensure lesion adequacy. Three weeks postprocedure, the LVOT gradient decreased to 38 mm Hg, and the patient’s condition improved to NYHA I. However, at the 4-year follow-up, the LVOT gradient increased to 60 mm Hg, and she reported NYHA II symptoms (Figures 2A, 2D, 2G).

### Case 2

A 68-year-old woman had NYHA III dyspnea and syncope, without a family history of HOCM. SAA had failed. The ECG was normal, and TTE revealed normal LVEF, IVS WT of 21 mm, and an LVOT gradient of 98 mm Hg at rest. The procedure was performed using the CartoSound® (Johnson & Johnson MedTech, New Brunswick, NJ) system, delivering 50 W with a contact force of 10–30*g* on the basal and mid-septal wall for 30 seconds per lesion, with an average impedance drop of 10 Ohms. Noncapture of pacing was observed, confirming effective ablation at each site. At 3 weeks, the LVOT gradient decreased to 20 mm Hg, with symptomatic improvement to NYHA I. However, at 4 years, the gradient increased to 70 mm Hg, and she reported NYHA III symptoms (Figures 2B, 2E, 2H).

### Case 3

A 21-year-old woman with a family history of HOCM in her father presented with NYHA III dyspnea. ECG indicated left ventricular hypertrophy with a Sokolow score of 44. TTE demonstrated normal LVEF, IVS WT of 29 mm, and an LVOT gradient of 126 mm Hg. Coronary angiography indicated inadequate septal anatomy for alcohol ablation, and the patient declined myectomy. ERASH was performed, using the EnSite Precision 3D mapping system, with an average power of 50 W delivered to the basal and mid-septal wall for 30 seconds per lesion. During the procedure, a right ventricular perforation with tamponade occurred, requiring emergency surgery. Post-ERASH, the LVOT gradient decreased to 13 mm Hg, and the patient improved to NYHA I. At the 4-year follow-up, the gradient remained stable at 7 mm Hg (Figures 2C, 2F, 2I, and Videos 1 and 2).

## Discussion

The European Society of Cardiology and American Heart Association/American College of Cardiology guidelines recommend septal reduction therapy in patients with symptomatic LVOT obstruction, with myectomy being preferred over SAA.^2^^,^^3^ Our case series demonstrates that ERASH is a viable alternative to SAA.

We used a retrograde transaortic approach for 3D mapping and LV septal ablation in all patients under ICE guidance. ICE enabled visualization of septal-anterior mitral contact (SAM) and hypertrophic zones, whereas 3D mapping identified low-voltage areas in the septum. The SAM contact zones and hypertrophic regions were ablated. Lesion efficacy was validated via noncapture of pacing, aiming for at least a 50% reduction in LVOT gradient. Ablation times ranged from 28 to 42 minutes, with 33 to 36 lesions applied per patient (Table 1).

**Table 1:** Summary of cases

Patient	Age	Sex	Procedure Summary	LVOT Gradient (Pre-ERASH)	LVOT Gradient (3 weeks Post-ERASH)	LVOT Gradient (4 years Post-ERASH)	NYHA Class (Pre-ERASH)	NYHA Class (4 years Post-ERASH)	No. of Lesions	Ablation Time
1	39	F	EnSite Precision™, 50 W, 30 s	147 mm Hg	38 mm Hg	60 mm Hg	III	II	36	33.6 min
2	68	F	Carto® 3 System, 50 W, 30 s, 10-30 g	98 mm Hg	20 mm Hg	70 mm Hg	III	III	35	32.8 min
3	21	F	EnSite Precision™, 50 W, 30 s	126 mm Hg	13 mm Hg	7 mm Hg	III	I	33	28.5 min

ERASH = endocardial radiofrequency ablation of septal hypertrophy; LVOT = left ventricular outflow tract.

None of the patients showed significant changes in IVS thickness. At the 21-day follow-up, TTE showed a 74% to 90% reduction in LVOT gradients (Table 2). We measured regional longitudinal strain (LS) and circumferential strain (CS) in the basal anteroseptal segment using vector velocity imaging. At 21 days post-ERASH, LS in the anteroseptal segment remained decreased compared with baseline, and regional CS increased above reference values (Table 2). At the 4-year follow-up, LVOT gradient reduction and LS remained in case 3, whereas the other 2 patients exhibited increasing LVOT gradients and LS, particularly in case 1. Regional CS remained elevated in all 3 cases (Table 2).

**Table 2:** Echocardiographic and cardiopulmonary exercise test characteristics of the patients

	Patient 1			Patient 2			Patient 3		
	Baseline	21 Days Post ERASH	4 Years Post ERASH	Baseline	21 Days Post ERASH	4 Years Post ERASH	Baseline	21 Days Post ERASH	4 Years Post ERASH
Septum thickness (mm)	22	22	21	21	19.9	19.3	29	30	25
LVOT -PG	147	38	60	98	20	70	126	13	7
Basal anteroseptal LS (%)	–14	–7	–15	–14	–11	–6	–8.3	–4	–4
Basal anteroseptal CS (%)	–13.7	–32	–28	–11	–28	–25	–22	–34	–29

CS = circumferential strain; ERASH = endocardial radiofrequency ablation of septal hypertrophy; LS = longitudinal strain; LVOT-PG = Left ventricular outflow tract peak gradient.

In the short term, our findings align with previous studies showing acute improvements in myocardial mechanics after septal ablation.^5^, ^6^^,^^7^ We observed significant decreases in regional LS, accompanied by compensatory increases in CS, suggesting a shift in myocardial contraction mechanics to maintain overall function. However, long-term outcomes were less predictable, with 2 patients showing recurrence of elevated LVOT gradients at the 4-year follow-up.

ERASH provides an effective method for reducing LVOT gradients in HOCM patients and offers a viable alternative to SAA, particularly in patients unsuitable for alcohol ablation. Although short-term improvements in LVOT gradients and myocardial strain are promising, long-term outcomes are less predictable, with some patients experiencing recurrence of LVOT obstruction.

Further studies are needed to assess the durability of ERASH in terms of mechanical remodeling, perfusion dynamics, and functional capacity, as measured by cardiorespiratory fitness parameters such as peak oxygen uptake and the ventilatory efficiency slope.

## Disclosures

The authors have no conflicts of interest to disclose.
