# Supplementary material for: Left ventricular mechanics analysis after endocardial radiofrequency ablation for hypertrophic obstructive cardiomyopathy
Source: HeartRhythm Case Rep. 2024 Nov 17;11(3):193–7. doi: 10.1016/j.hrcr.2024.11.009 (PMC11963089; doi:10.1016/j.hrcr.2024.11.009)
Supplement: Video Legends [file mmc3.docx]

**Video 1.** Case 3. Pre ERASH Apical three-chamber view transthoracic echocardiography showing LV hypertrophy with normoquinesia and normal ejection fraction**.**

**Video 2.** Case 3. Post ERASH Apical three-chamber view transthoracic echocardiography with no evident changes in LV contraction.
